# Acetazolamide Improves Right Ventricular Function and Metabolic Gene Dysregulation in Experimental Pulmonary Arterial Hypertension

**DOI:** 10.3389/fcvm.2021.662870

**Published:** 2021-06-17

**Authors:** Fotios Spyropoulos, Zoe Michael, Benjamin Finander, Sally Vitali, Kosmas Kosmas, Panagiotis Zymaris, Brian T. Kalish, Stella Kourembanas, Helen Christou

**Affiliations:** ^1^Department of Pediatric Newborn Medicine, Brigham and Women's Hospital, Boston, MA, United States; ^2^Division of Newborn Medicine, Boston Children's Hospital, Boston, MA, United States; ^3^Harvard Medical School, Boston, MA, United States; ^4^Department of Neurobiology, Harvard Medical School, Boston, MA, United States; ^5^Department of Anesthesia and Critical Care Medicine, Boston Children's Hospital, Boston, MA, United States

**Keywords:** carbonic anhydrase inhibitor, cardiac metabolism, fatty acid oxidation, right heart failure, Sugen 5416 hypoxia model

## Abstract

**Background:** Right ventricular (RV) performance is a key determinant of mortality in pulmonary arterial hypertension (PAH). RV failure is characterized by metabolic dysregulation with unbalanced anaerobic glycolysis, oxidative phosphorylation, and fatty acid oxidation (FAO). We previously found that acetazolamide (ACTZ) treatment modulates the pulmonary inflammatory response and ameliorates experimental PAH.

**Objective:** To evaluate the effect of ACTZ treatment on RV function and metabolic profile in experimental PAH.

**Design/Methods:** In the Sugen 5416/hypoxia (SuHx) rat model of severe PAH, RV transcriptomic analysis was performed by RNA-seq, and top metabolic targets were validated by RT-PCR. We assessed the effect of therapeutic administration of ACTZ in the drinking water on hemodynamics by catheterization [right and left ventricular systolic pressure (RVSP and LVSP, respectively)] and echocardiography [pulmonary artery acceleration time (PAAT), RV wall thickness in diastole (RVWT), RV end-diastolic diameter (RVEDD), tricuspid annular plane systolic excursion (TAPSE)] and on RV hypertrophy (RVH) by Fulton's index (FI) and RV-to-body weight (BW) ratio (RV/BW). We also examined myocardial histopathology and expression of metabolic markers in RV tissues.

**Results:** There was a distinct transcriptomic signature of RVH in the SuHx model of PAH, with significant downregulation of metabolic enzymes involved in fatty acid transport, beta oxidation, and glucose oxidation compared to controls. Treatment with ACTZ led to a pattern of gene expression suggestive of restored metabolic balance in the RV with significantly increased beta oxidation transcripts. In addition, the FAO transcription factor peroxisome proliferator-activated receptor gamma coactivator 1-alpha (*Pgc-1*α) was significantly downregulated in untreated SuHx rats compared to controls, and ACTZ treatment restored its expression levels. These metabolic changes were associated with amelioration of the hemodynamic and echocardiographic markers of RVH in the ACTZ-treated SuHx animals and attenuation of cardiomyocyte hypertrophy and RV fibrosis.

**Conclusion:** Acetazolamide treatment prevents the development of PAH, RVH, and fibrosis in the SuHx rat model of severe PAH, improves RV function, and restores the RV metabolic profile.

## Introduction

Pulmonary arterial hypertension (PAH) is a progressively debilitating and ultimately fatal chronic disorder. Patients are often diagnosed at advanced stages of the disease and develop worsening exertional shortness of breath that seriously compromises their quality of life. The pathophysiology of PAH involves pulmonary vasoconstriction and vascular remodeling that promote right ventricular (RV) heart hypertrophy and failure (1). RV function is recognized as the most important prognostic factor (2), and RV failure (RVF) is the principal cause of death in PAH. Despite our improved understanding of the molecular and pathogenetic mechanisms of the disease (3–5), state-of-the-art therapies currently provide only modest improvements in quality of life, and thus, there is an unmet clinical need to develop novel therapies. The primary mechanism targeted by current therapies is vasoconstriction, while therapies that improve RV function do not exist (6). While there has been progress in our understanding of the molecular basis of RVF in PAH, little to no progress has been made in translating this knowledge to clinical therapies (7). In order to improve clinical outcomes in PAH, we must leverage our improved understanding of the pathogenesis of RVF to develop novel therapeutic strategies that directly or indirectly improve RV function (8). In the adult heart, most of the energy requirements (>95%) are provided through mitochondrial oxidative phosphorylation of lipids/fatty acids (FAs) and glucose. FA oxidation (FAO) accounts for 50–70% of energy production, and only about 30% is derived from glucose oxidation (GO) (9). In PAH, RVF is characterized by decreased FAO and increased glycolysis (10). This phenomenon has been described in PAH patients and preclinical models (9, 11–17). Nevertheless, the pathobiological mechanisms that account for this effect are unknown, and specific pharmacotherapies targeting the dysregulated RV metabolism have not been developed.

Carbonic anhydrases (CAs) are ubiquitously expressed enzymes that catalyze the enzymatic hydration of carbon dioxide to bicarbonate and protons. CAs facilitate GO (18), and evidence suggests that CA inhibitors (CAIs) suppress GO and promote FAO (19). Importantly, CAIs are effective in left heart failure, as they improve left ventricular (LV) systolic function by echocardiography *in vivo* (20) and prevent cardiomyocyte hypertrophy *in vitro* (21). However, their effect on RV function in PAH has not been studied.

In the well-established Sugen 5416/hypoxia rat model of PAH (SuHx-PAH), a clinically relevant rodent model, the remodeled RV demonstrates a gene expression profile consistent with a multilevel impairment of FA metabolism (16). This includes increased glycolysis, decreased FAO, and mitochondrial dysfunction. We hypothesized that in SuHx-PAH, CA inhibition with acetazolamide (ACTZ) will promote FAO to restore FA utilization and improve RV function. In this manuscript, we report that treatment with ACTZ improves all aspects of FA transport and FAO and ameliorates adverse RV remodeling.

## Materials and Methods

### Animal Model

Adult (12-week-old) male Sprague–Dawley rats (250–300 g) were purchased from Charles River Laboratories (Wilmington, MA) and acclimatized for 2–3 days prior to the experiments. PAH was induced as previously described (22) with a subcutaneous injection of 20 mg/kg Sugen 5416 (Sigma, St. Louis, MO) in dimethyl sulfoxide (DMSO; Sigma, St. Louis, MO), placement in hypoxia (9% O_2_) for 3 weeks, and return to normoxia. Oxygen was controlled to 9% ± 0.2% by an OxyCycler controller (BioSpherix, Redfield, NY), and ventilation was adjusted with a fan and port holes to remove CO_2_ and ammonia. Control rats were injected with an equal volume of vehicle (DMSO) in normoxia. The endpoints of the study were 24 days after injection (Supplementary Figure 1).

### Treatment Protocol

Rats were randomized into control and ACTZ treatment groups. ACTZ (Spectrum, Gardena, CA) (1.7 mg/ml) was added to the drinking water. Sucrose (5% w/v) was added to treated and control animals to increase water intake. Water consumption was monitored and estimated to be ~20 ml per rat per diem or ~100 mg/kg/day. The treatment protocol consisted of administration of ACTZ from days 7 to 24. Experimental groups: Ctrl (normoxia control with vehicle injection), SuHx (Sugen/Hypoxia), SuHx+ACTZ (Sugen/Hypoxia treated with ACTZ; Supplementary Figure 1).

### Hemodynamic and Ventricular Hypertrophy Measurements

Hemodynamic measurements were performed as previously described (23). Animals were anesthetized with inhalation of 3% isoflurane, intubated through a tracheotomy, and mechanically ventilated on a rodent ventilator (Harvard Apparatus, tidal volume 1 ml/100 g body weight, 60 breaths per minute). The thoracic cavity was opened by incision of the diaphragm. A 23-gauge butterfly needle with tubing attached to a pressure transducer was inserted first into the right ventricle and then into the left ventricle, and pressure measurements were recorded with PowerLab monitoring hardware and software (ADInstruments, Colorado Springs, CO). Mean RV systolic pressure (RVSP) and LV systolic pressure (LVSP) (in mmHg) over the first 10 stable heartbeats were recorded. Mean pulmonary artery pressure (mPAP) for all experimental animals was calculated using our previously published and validated formula (mPAP = 0.53 × RVSP + 2.3) (*r*^2^ = 0.92) (22). RV hypertrophy (RVH) was assessed by weighing RV mass and expressed as Fulton's index (FI, ratio of RV weight to the LV+septal weight) or as the ratio of RV weight to total body weight (RV/BW).

### Lung and Heart Histology and Morphometric Analysis

In a subset of experimental animals, the lungs were inflated by perfusing the trachea with cold 4% paraformaldehyde (PFA), excised, and fixed in 4% PFA overnight at 4°C followed by paraffin embedding. Lung sections (6 μm) were stained with hematoxylin and eosin (H&E) and examined with light microscopy. Images of the arterioles were captured with a microscope digital camera system (Nikon) and analyzed using ImageJ (NIH, USA). Arterioles of comparable size (50–100 μm diameter) per rat from the lungs of 5–6 different rats from each experimental group were evaluated. The percent wall thickness was determined by dividing the area occupied by the vessel wall by the total cross-sectional area of the arteriole as previously reported (4). This method accounts for uneven vessel wall thickness and areas that have obliquely sectioned pulmonary arterioles. In a subset of animals, the heart was stopped in diastole with a KCl injection and the heart was fixed in formalin, embedded in paraffin, and the RV free wall was sectioned longitudinally. Cardiomyocyte hypertrophy was evaluated in H&E-stained sections by measuring the cellular diameter at the level of the nucleus as previously described (24). We used Masson's trichrome stain to detect collagen deposition and quantify interstitial and perivascular fibrosis using ImageJ software as previously described (25). All analyses were performed in a blinded fashion to the experimental groups.

### Echocardiography

Transthoracic 2D M-mode and Doppler images were acquired at the Brigham and Women's Hospital's Cardiovascular Physiology Research Core facility with a Visual Sonics 3100 ultrasound system equipped with an MX250 (13–24 MHz) probe as previously described (26). In brief, animals were lightly anesthetized with isoflurane (1–3%) titrated to maintain a minimum heart rate of 300/min while they continued to breathe spontaneously for the duration of the procedure. Pulmonary hemodynamics were assessed by measurement of pulmonary artery acceleration time (PAAT), pulmonary artery ejection time (ET), and the PAAT/ET ratio to account for heart rate variability. To assess RV morphology and function, the following measures were obtained using M-mode: end-diastolic RV free wall thickness (RVFWTd), end-diastolic RV diameter (RVEDD), and derived tricuspid annular plane systolic excursion (TAPSE). Images were analyzed with Vevo-Lab software (V.3.1.1 FUJIFILM Visualsonics, Toronto, Canada). The sonographer and the analyzer were both blinded to the experimental groups.

### Isolation of mRNA and Transcript Expression Analysis

RNA isolation, analysis, and reporting followed minimum information for publication of quantitative real-time polymerase chain reaction (PCR) experiments (MIQE) criteria (27). Cardiac tissues were lysed with TRIzol reagent (Invitrogen) per manufacturer's instructions and homogenized. RNA quantity and quality were assessed with NanoDrop 2000c spectrophotometer (NanoDrop Technologies). Complementary DNA was generated with the SuperScript III First-Strand Synthesis System (Invitrogen). Quantification of mRNA transcript levels was performed with StepOnePlus RT-PCR cycler (Applied Biosystems) using iTaq Universal SYBR Green Supermix (Bio-Rad, Hercules, CA). Gene-specific primers for all transcript variants were designed across exon–exon junctions when possible and ordered through Integrated DNA Technologies (see Supplementary Table 1 for primer sequences). Nucleoporin 133 (Nup133) was used as a housekeeping gene for normalization. Expression was analyzed with the comparative Ct method (28).

### RNA Sequencing

The RV free wall was dissected and snap frozen in liquid nitrogen. RNA extraction and quantification and sample integrity were performed by Novogene using Nanodrop spectrophotometer and Agilent 2100 bioanalyzer, respectively. Library preparation and sequencing were performed using an Illumina Platform PE150 by Novogene. Fastq files obtained from paired-end sequencing were aligned to the Rattus Norvegicus 6.0 ENSEMBL genome using standard parameters in STAR version 2.5.4a. Raw count files were generated next using Htseq version 0.9.1 with –t exon option enabled to ensure transcript specificity. Differential gene expression was performed using DESeq2 version 1.26.0 using standard parameters. Counts were normalized using a negative binomial distribution as is standard with DESeq2. Experimental and control groups were compared using a Wald test in a pairwise comparison. Only genes with a multiple-comparison adjusted *P* < 0.05 were considered significant in the comparison. Gene Ontology analysis was performed using the PANTHER classification system and a statistical overrepresentation test on significantly changed genes.

### Western Blotting

Western blot analysis was performed as previously described (4). Briefly, RVs were homogenized in radioimmunoprecipitation assay (RIPA) lysis buffer containing cOmplete™ protease inhibitor (Roche, Indianapolis, IN), and protein concentration was determined by Pierce BCA protein assay kit (ThermoFisher). Proteins were separated by 12% Tris SDS polyacrylamide gel electrophoresis and transferred to nitrocellulose membranes (Bio-Rad) using a semi-dry system (Bio-Rad). Membranes were blocked with 5% w/v non-fat dry milk, incubated with primary antibodies (4°C, overnight), and incubated with secondary antibodies at room temperature for 1 h. Proteins were detected by horseradish peroxidase (HRP) chemiluminescence, and lanes were quantified using ImageJ analysis software. An antibody against ACADM (1:10,000; ERP3708) was purchased from Abcam. The vinculin antibody (1:1,000; E1E9V) was purchased from Cell Signaling and was used as a loading control.

### Statistical Analysis

Statistical analyses were performed using GraphPad Prism (GraphPad Software, La Jolla, CA). We used one-way ANOVA with Tukey's posttest (when comparing multiple groups) or Student's t-test (when comparing two groups). Where numbers permitted, we used the D'Agostino and Pearson omnibus normality test, and data with non-Gaussian distribution were analyzed by Kruskal–Wallis test with Dunn's posttest or Mann–Whitney U test. Data are presented as individual data points and mean with standard deviation (SD) or as mean with standard error of the mean (SEM). Individual statistical tests are described in the corresponding figure legends. *P* < 0.05 were considered statistically significant.

## Results

### Transcriptional Signature of Right Ventricular Hypertrophy in Sugen 5416/Hypoxia-Induced Pulmonary Hypertension

**Figure 1:**
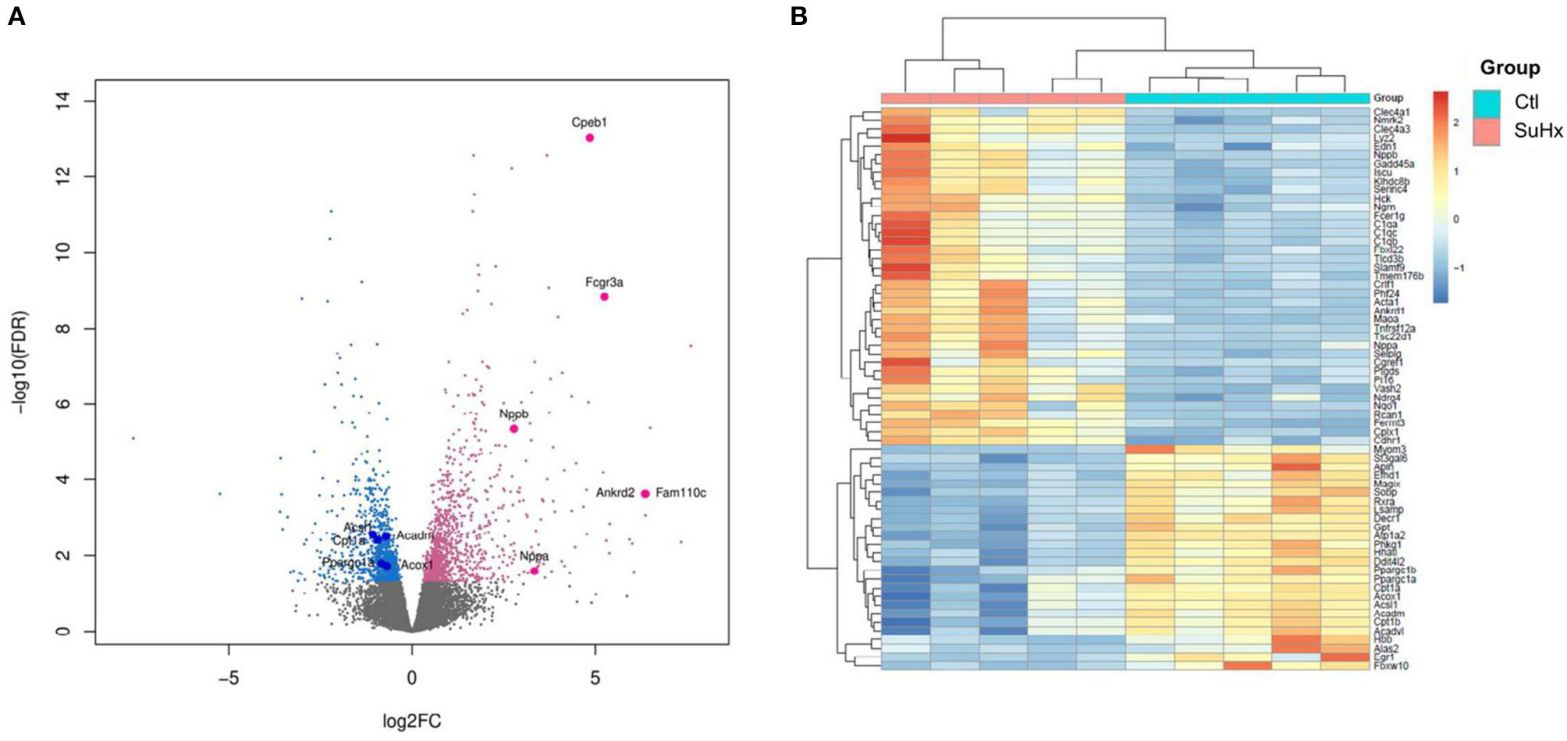
Differential expression of RV genes reveals a distinct signature between Sugen 5416/hypoxia (SuHx) and control (Ctl) groups. **(A)** Volcano plot of the cardiac mRNA transcripts of the RV in the SuHx vs. Ctl groups. Significantly downregulated genes are in blue, significantly upregulated genes are in pink, nonsignificant genes are in gray. Highlighted, upregulated classic markers of ventricular hypertrophy and failure (*Nppa, Nppb*), downregulated genes involved in fatty acid metabolism (*Ppargc1a Cpt1a, Acadm, Acox1*, and *Acsl1*), and upregulated genes that were used for RNA sequencing validation (*Ankrd2, Fam110c, FCGR3a, and Cpeb1*). **(B)** *Z*-score heatmap of 65 significantly differentially expressed genes related to metabolic pathways between SuHx and control groups, *n* = 5 individual animals per group. For experimental design (see Supplementary Figure 1).

**Figure 2:**
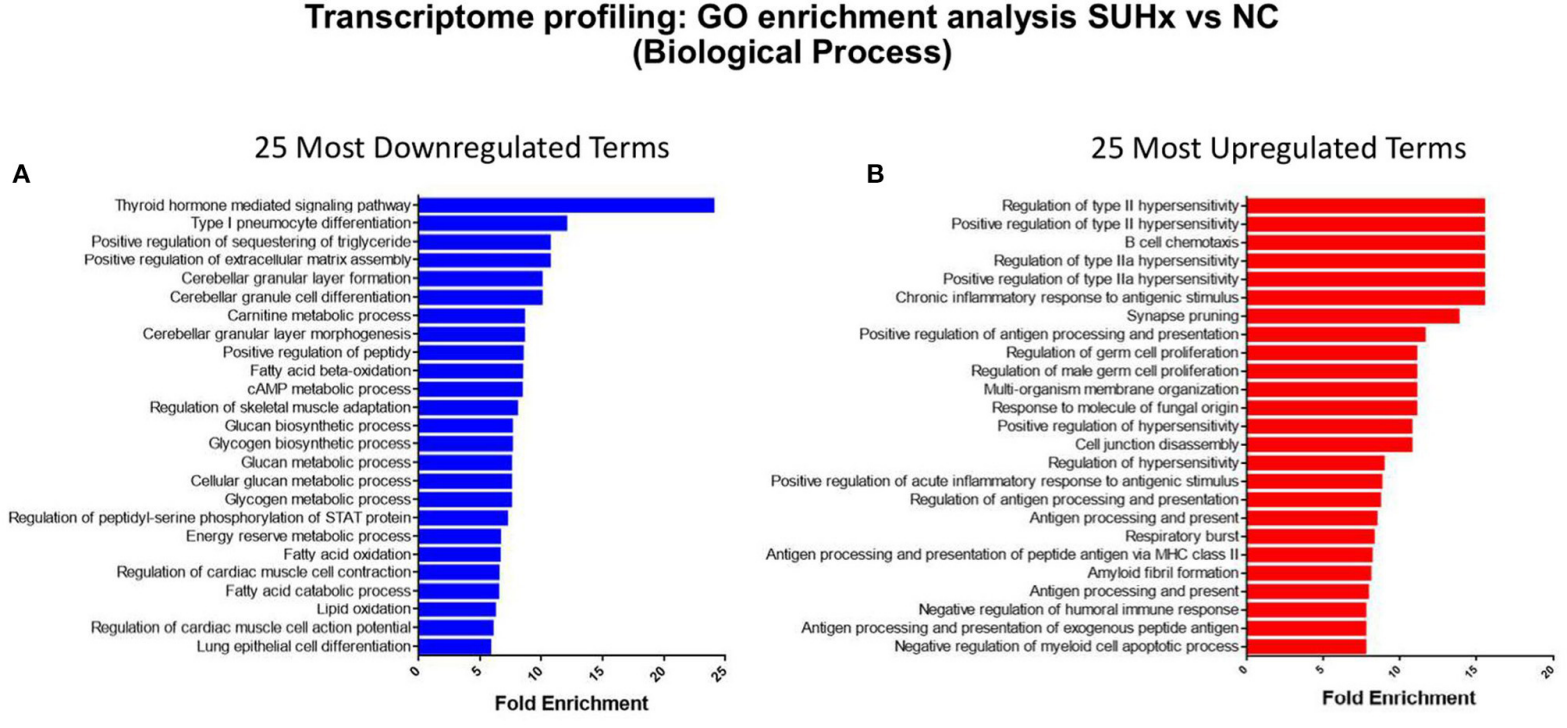
Gene ontology enrichment analysis of biological processes in the RV transcripts between Sugen 5416/hypoxia (SuHx) and control groups. **(A)** The top 25 most significantly downregulated and **(B)** the top 25 most significantly upregulated terms by false discovery rate (FDR).

We used RNA sequencing to characterize the transcriptomic profile of the RV and evaluate differential expression of genes during SuHx-induced RVH at an early time point, 24 days after Sugen injection and hypoxic exposure. There was a distinct transcriptomic signature in the RV of SuHx animals compared to controls, with 1,129 upregulated and 892 downregulated genes with a false discovery rate (FDR) <5% (Figure 1; Supplementary Figure 2). We subsequently performed quantitative PCR (qPCR) on a different cohort of animals to validate the RNA sequencing data. We successfully validated four of the transcripts that were among the most significantly and differentially upregulated (Table 1). We then proceeded with gene ontology analysis that revealed that the most upregulated transcripts were related to inflammatory pathways, cell proliferation, and cytoskeleton organization and that the most downregulated pathways were related to mitochondrial function and metabolic processes including fatty acid oxidation (FAO) and lipid metabolism (Figure 2; Data Sheets 1–6 in Supplementary Material). When we evaluated transcripts involved in FAO, most significantly changed (FDR <5%) transcripts were downregulated (Supplementary Figure 3), suggesting a metabolic switch with decreased FAO.

**Table 1:** Validation of RNA sequencing.

**Gene**	**Pathway**	**RNA sequencing**	**PCR validation**		
		**Fold change**	**Mean FC**	**SEM**	***p-*value**
*Ankrd2*	Muscle differentiation	82.82346937	6.3222	1.378	0.0157
*Fam110c*	Cell migration	81.57599817	4.471	0.63	0.004
*Fcgr3a*	Tissue remodeling	38.00645515	14.15	2.066	<0.0001
*Cpeb1*	Cell migration	28.80605667	4.649	0.6437	<0.0001

*Top differentially expressed genes in the RV of SuHx vs. control rats validated by qPCR in an independent cohort of animals (n = 5 per group)*.

### Treatment With Acetazolamide Prevents Pulmonary Hypertension and Vascular Remodeling in Sugen 5416/Hypoxia-Induced Pulmonary Hypertension

**Figure 3:**
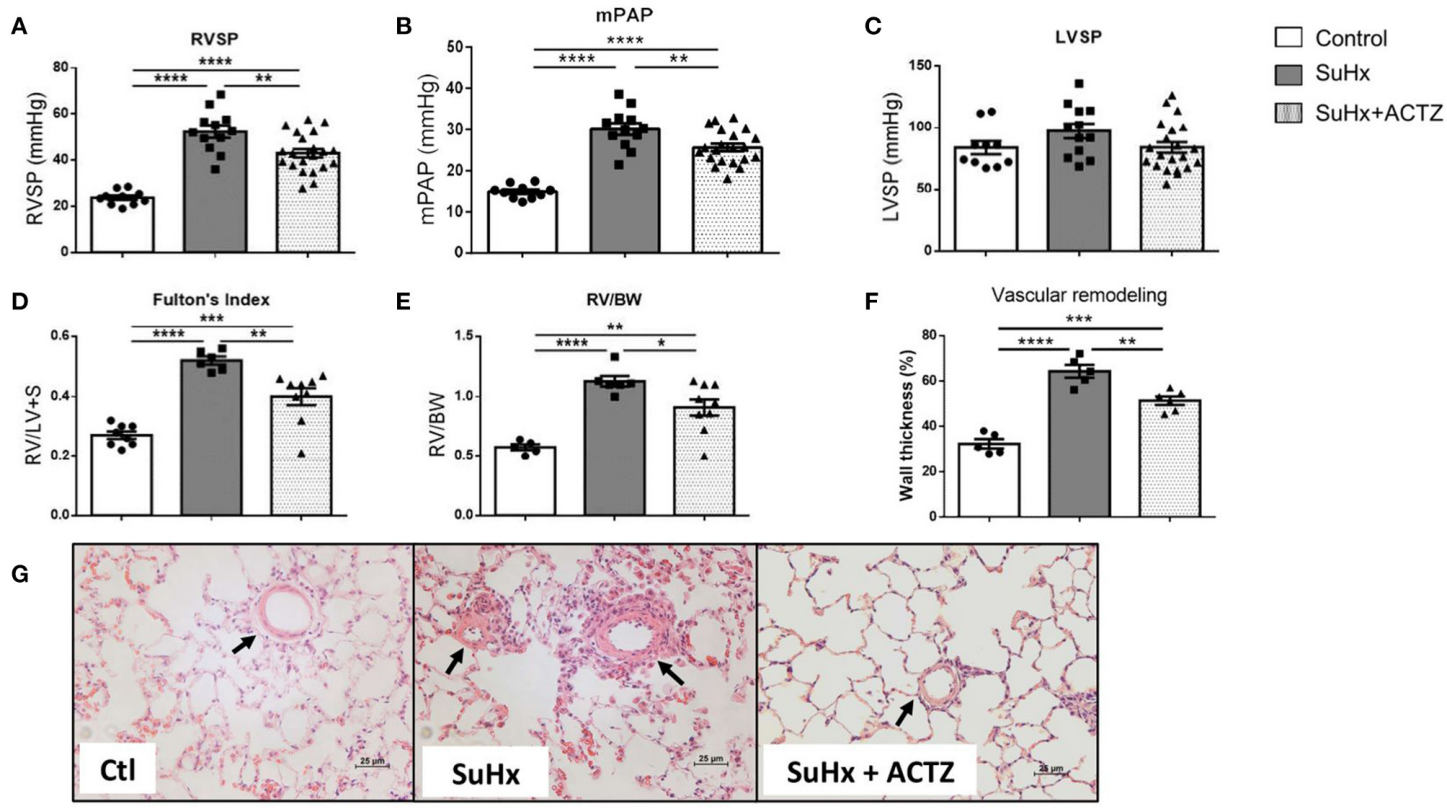
Treatment with acetazolamide (ACTZ) ameliorates Sugen 5416/hypoxia (SuHx)-induced pulmonary hypertension (PH). Improved hemodynamic parameters with ACTZ treatment **(A)** Right ventricular (RV) systolic pressure (RVSP), **(B)** mean pulmonary artery pressure (mPAP), and **(C)** left ventricular systolic pressure (LVSP) in mmHg in the three experimental groups [control (Ctl), SuHx, and SuHx+ACTZ] (*n* = 10–21 animals per experimental group). Amelioration of RV hypertrophy (RVH) with ACTZ treatment, **(D)** Fulton's index (FI), and **(E)** RV-to-body weight ratio (*n* = 6–9 animals per experimental group) (FI, ratio of RV weight to left ventricular+septal weight). Amelioration of pulmonary vascular remodeling after treatment with ACTZ. **(F)** Morphometric analysis of pulmonary vascular remodeling assessed by % wall thickness. *n* = 6–15 arterioles (diameter 50–100 μm) from *n* = 5–6 animals per group. **(G)** Representative images of pulmonary arterioles on H&E-stained lung sections of Ctl, SuHx, and SuHx animals treated with ACTZ. Scale bars = 25 μm. Arterioles indicated by arrows. Data presented as mean ± SEM. Statistical analysis by one-way ANOVA and Tukey's *post-hoc* test. Error bars are mean ± SEM (*P < 0.05, **P < 0.01, ***P < 0.001, and ****P < 0.0001).

As we previously reported, early treatment with ACTZ (from days 7 to 24 after SuHx-Protocol, Supplementary Figure 1) led to significantly lowered RVSP and mPAP compared to those of the SuHx group (Figures 3A,B), without affecting the LVSP (Figure 3C). RVH as assessed by FI and RV/BW were significantly decreased in the SuHx/ACTZ group (Figures 3D,E). Histologic evaluation of peripheral lung sections showed that there was increased wall thickness of arterioles <100 μm in diameter in SuHx animals and a significant reduction in wall thickness in pulmonary arterioles in the ACTZ treatment group. Given the early time point in the disease course (3 weeks), we did not observe any occlusive or plexiform lesions, known to occur at 13–14 weeks after SuHx exposure (Figures 3F,G).

### Carbonic Anhydrase Inhibitor Acetazolamide Improves Right Ventricular Compliance and Function Without Compromising the Left Ventricular Function

**Figure 4:**
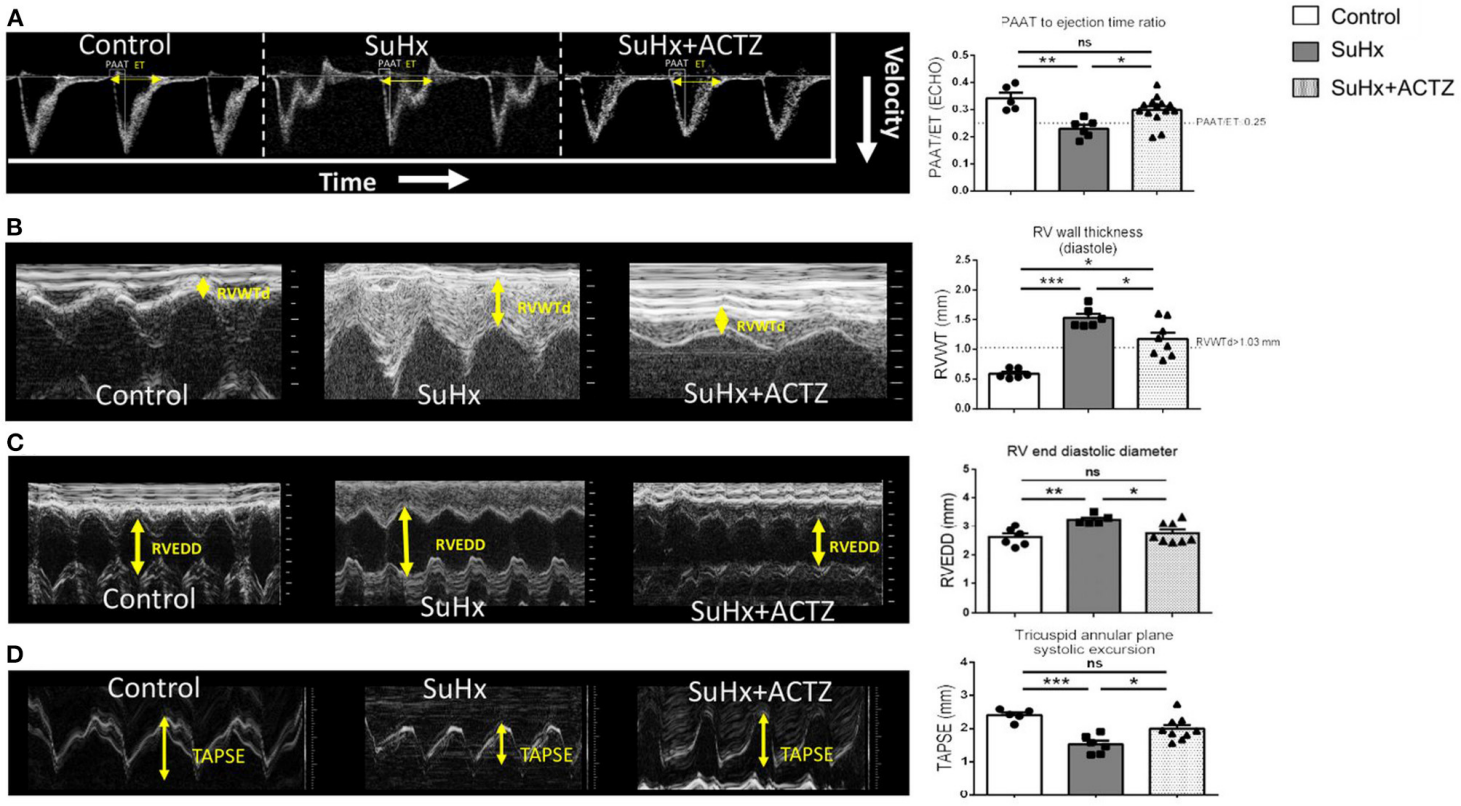
Improved echocardiographic parameters in Sugen 5416/hypoxia (SuHx)-induced pulmonary hypertension (PH) with acetazolamide (ACTZ) treatment. **(A)** Pulmonary artery acceleration time to ejection time ratio (PAAT/ET). Representative pulmonary artery Doppler waveform and quantitative data (*n* = 5–14 animals per group). **(B)** Right ventricular (RV) wall thickness in diastole (RVWT). Representative M-mode images from the right parasternal long axis imaging view and quantitative analysis (*n* = 6–8 animals per group). **(C)** RV end-diastolic diameter (RVEED). Representative M-mode images and quantitative data (*n* = 5–8 animals per group). **(D)** Tricuspid annular systolic excursion (TAPSE). Representative images and quantitative data (*n* = 5–9 animals per group). Experimental groups: Control, SuHx, and SuHx+ACTZ. Statistical analysis by one-way ANOVA and Tukey's *post-hoc* test. Error bars are mean ± SEM (*P < 0.05, **P < 0.01, and ***P < 0.001).

To evaluate the effects of ACTZ on the RV, we first assessed PAAT, which is a measure of PAP, vascular resistance, and RV compliance. As expected, SuHx animals had a significantly lower PAAT (Supplementary Figure 4) that significantly increased after ACTZ treatment and was similar to the control group. To normalize for heart rate variability, we used the ratio of PAAT to ET with similar results. The PAAT/ET ratio threshold of ≤0.25, which we have previously shown to be a reliable diagnostic marker of pulmonary hypertension (PH) (26), was consistently less or equal to 0.25 in all but one SuHx animal and was ≥0.25 in all controls and all but two ACTZ treatment animals (Figure 4A). Similarly, the echocardiographic marker of RVH—RV wall thickness (RVWT)—was significantly lower in the SuHx/ACTZ group compared to the SuHx group. Our previously validated RVWT cutoff value of >1.03 mm that reliably predicts RVH captured all SuHx animals. For the ACTZ treatment group, although there was improvement of RVH, it did not reach the baseline levels (Figure 4B). To evaluate RV function, we utilized RV end-diastolic diameter (RVEDd), which was significantly increased in the SuHx group and tricuspid annular plane systolic excursion (TAPSE), which was significantly decreased in the SuHx animals. ACTZ treatment restored these measures back to the normal values, as there was no difference with the control group, indicating significantly improved RV function (Figures 4C,D). Finally, we evaluated the effects of ACTZ treatment on the systemic circulation, and we did not observe any differences between groups in the LV function, as assessed by unchanged ejection fraction (EF), fractional shortening (FAC), and cardiac output (Supplementary Figures 5A–C). Similarly, there were no changes in indices of LV remodeling as assessed by LV end-diastolic volume and posterior and anterior wall thickness (Supplementary Figures 5D–F).

### Acetazolamide Prevents Development of Cardiomyocyte Hypertrophy and Right Ventricular Interstitial Fibrosis

**Figure 5:**
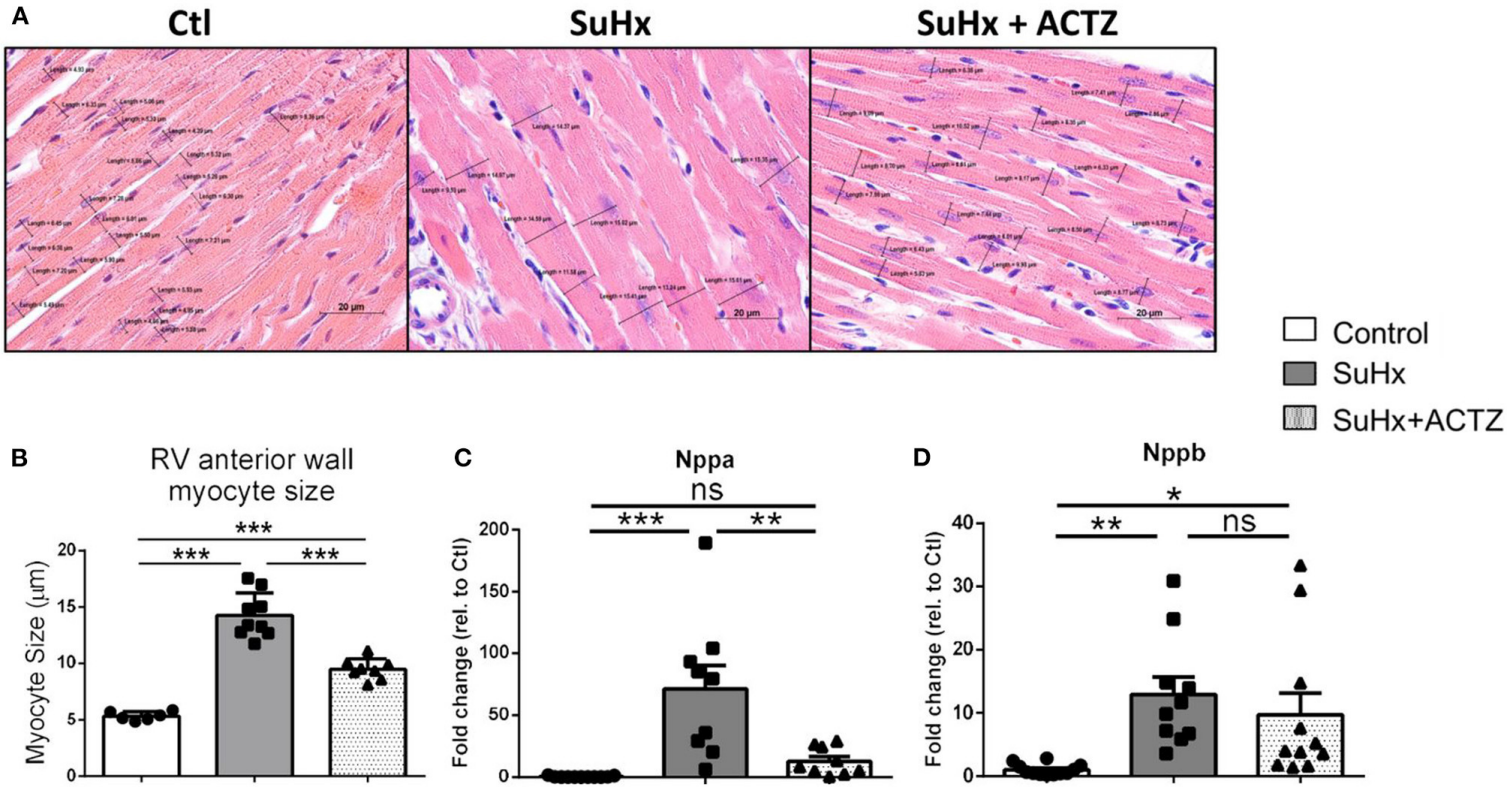
Amelioration of right ventricular (RV) cardiomyocyte hypertrophy in acetazolamide (ACTZ)-treated Sugen 5416/hypoxia (SuHx) animals. **(A)** Representative longitudinal RV free wall sections stained with hematoxylin and eosin. Black lines indicate cell diameters at the level of the nucleus. Scale bar 20 μm. **(B)** Quantitative analysis of myocyte size in the three experimental groups (*n* = 6–9 animals per group). Real-time PCR analysis of mRNA levels of the cardiac hypertrophy transcripts **(C)** atrial natriuretic peptide (*Nppa*) and **(D)** brain natriuretic peptide (*Nppb*) in RV from the experimental groups (*n* = 10–12 per group). Experimental groups: Control, SuHx, and SuHx+ACTZ. Statistical analysis by one-way ANOVA and Tukey's *post-hoc* test. Error bars are mean ± SEM. *P < 0.05, **P < 0.01, and ***P < 0.001.

**Figure 6:**
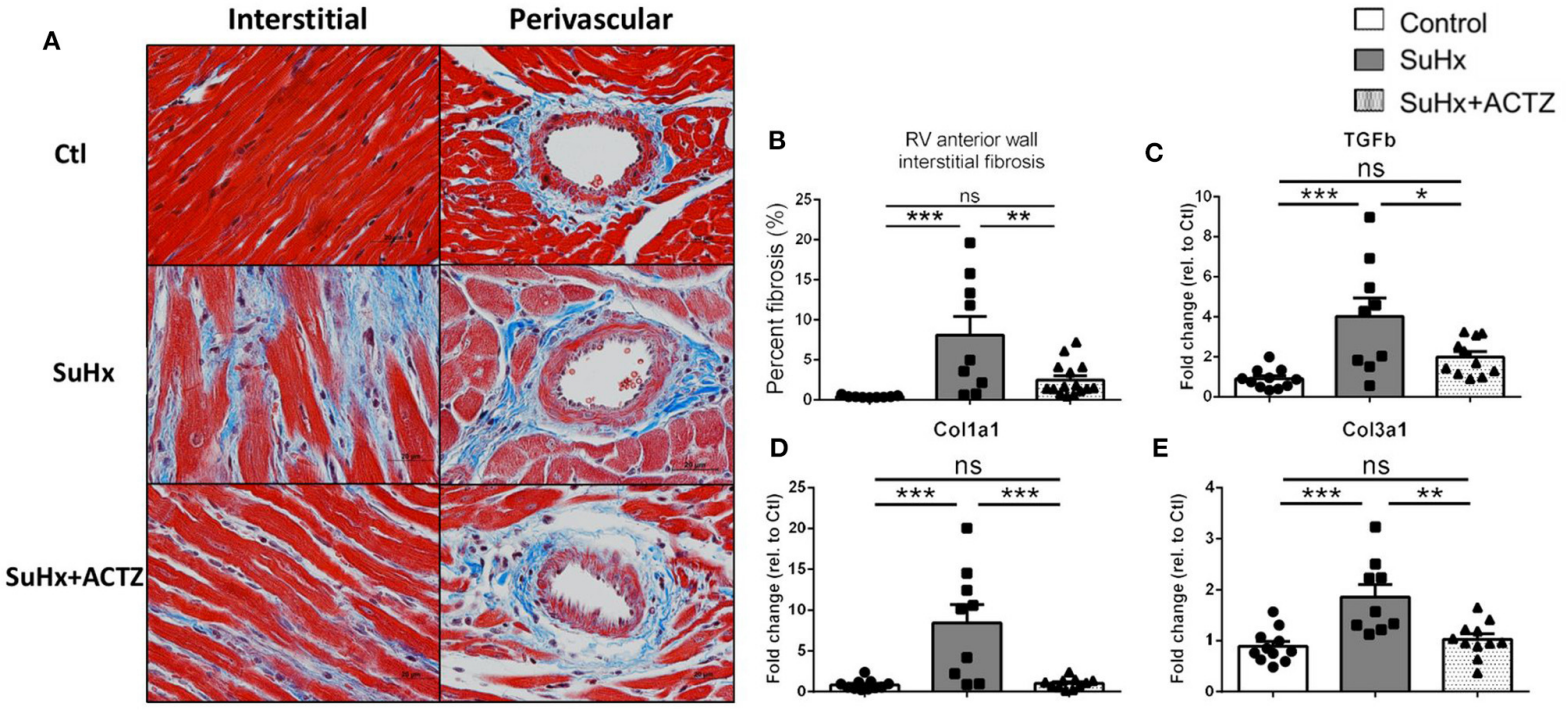
Amelioration of interstitial cardiac fibrosis in the right ventricle of Sugen 5416/hypoxia (SuHx) animals treated with acetazolamide (ACTZ). **(A)** Representative cardiac tissue sections stained with Masson's trichrome stain to detect interstitial and perivascular fibrosis. Scale bar 20 μm. **(B)** Quantitative analysis of interstitial fibrosis in the RV in the three experimental groups (*n* = 6–9 animals per group). Real-time PCR analysis of the fibrosis associated transcripts **(C–E)** transforming growth factor-β1(*Tgfb1*), collagen α-1(I) chain (*Col1a1*), collagen α-1(III) chain (*Col3a1*) (*n* = 10–12 per group). Experimental groups: Control, SuHx, and SuHx+ACTZ. Statistical analysis by one-way ANOVA and Tukey's *post-hoc* test. Error bars are mean ± SEM (*P < 0.05, **P < 0.01, and ***P < 0.001; ns, nonsignificant).

Cardiomyocyte hypertrophy as assessed by analysis of H&E-stained sections from RVs demonstrated that ACTZ treatment prevented hypertrophy at the cardiomyocyte level (Figures 5A,B). We then assessed the classic markers of cardiac hypertrophy and failure, atrial natriuretic peptide (ANP, *Nppa*), and brain natriuretic peptide (BNP, *Nppb*), which were found to be significantly elevated in the SuHx group by RNA sequencing (RNA-seq), and these were again validated to be significantly increased in the SuHx animals of this cohort. We found significantly decreased *Nppa* mRNA levels and a nonsignificant trend of decreased *Nppb* mRNA levels in the RVs in the SuHx/ACTZ group compared to the SuHx group (Figures 5C,D). We also noted increased levels of RV interstitial fibrosis in the SuHx group as assessed by Masson's trichrome staining, and this was significantly decreased in the SuHx/ACTZ group (Figures 6A,B). We did not observe any difference in perivascular fibrosis in between groups. We also evaluated RV expression of several markers of fibrosis (*Tgfb1, Col1a1*, and *Col3a1*), and all were significantly elevated in the SuHx animals and were attenuated by treatment with ACTZ (Figures 6C–E).

### Acetazolamide Partially Restores the Dysregulated Metabolic Profile of the Right Ventricle in Sugen 5416/Hypoxia-Induced Pulmonary Hypertension

**Figure 7:**
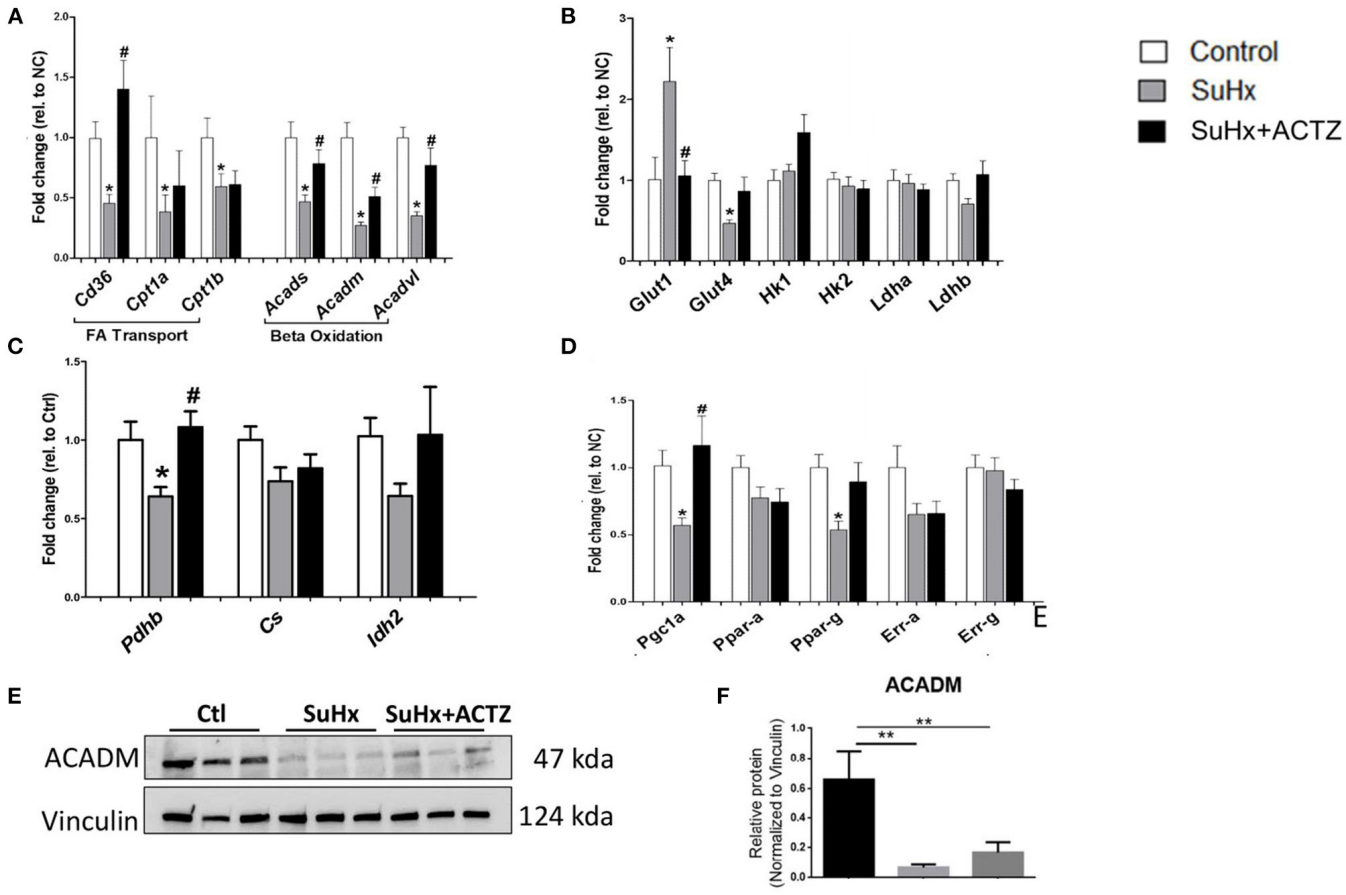
Effects of acetazolamide (ACTZ) treatment on right ventricular (RV) metabolism associated markers in Sugen 5416/hypoxia (SuHx) animals. **(A)** Fatty acid transport and oxidation transcripts. **(B)** Glucose uptake and glycolysis markers. **(C)** Tricarboxylic acid cycle enzymes. **(D)** Mitochondrial transcription factors. **(E)** Representative immunoblots for beta oxidation enzyme medium-chain specific acyl-CoA dehydrogenase (ACADM) and **(F)** quantitative analysis of ACADM protein levels in the three experimental groups (*n* = 3 per group). Experimental groups: Control, SuHx, and SuHx+ACTZ. Statistical analysis by one-way ANOVA and Tukey's *post-hoc* test. Error bars are mean ± SEM (*P < 0.05, **P < 0.01 compared to control, and ^#^*P* < 0.05 compared to SuHx).

Metabolic switching with suppression of beta oxidation is one of the hallmarks of RVF, which was also supported by our RNA-seq data set in the SuHx group. Validation of the RNA-seq data in a separate cohort of animals by real-time PCR showed that three key genes involved in fatty acid transport including cell membrane transport (*Cd36*) and mitochondrial transport (*Cpt1a* and *Cpt1b)* were significantly downregulated in the right ventricles of SuHx animals, and ACTZ treatment restored the expression of *Cd36* but had no effect on *Cpt1a* and *b*. Similarly, we confirmed the downregulation of FAO transcripts, including Acetyl-CoA dehydrogenases for small, medium, and large FA in right ventricles of SuHx animals, and ACTZ treatment significantly upregulated the expression all three enzymes (Figure 7A). This downregulation of *Acadm* in SuHx was also seen at the protein level, and there was a trend of increased expression with ACTZ treatment (Figures 7E,F). We then assessed transcription factors that regulate FAO. Peroxisome proliferator-activated receptor gamma coactivator 1-alpha (*Pgc-1*α) was significantly downregulated in the SuHx group, as observed in the RNA-seq data set and confirmed in this cohort, and ACTZ treatment increased its expression levels back to baseline. Peroxisome proliferator-activated receptor (*Ppar*) gamma (*Ppar-*γ) showed a similar trend, and there were no differences on *Ppar-*α, estrogen-related receptor (Err) alpha, *Err-a, and Err-*γ (Figure 7D).

We subsequently evaluated markers of glycolysis and found that glucose transporter 1 (*Glut1*) was significantly upregulated in the SuHx group, and ACTZ treatment decreased its expression to baseline levels. Insulin-regulated glucose transporter 4 (*Glut 4*) was downregulated in the SuHx rats, and there was a trend toward increased expression with ACTZ treatment. No significant differences were found in the rest of the glycolytic transcripts between the three groups, including hexokinase I and II (*HkI, HkII*) and lactate dehydrogenases A and B (*Ldha, Ldhb*), similar to the transcriptomic data (Figure 7B). We then assessed transcripts involved in the citric acid cycle and found significant downregulation of pyruvate dehydrogenase b (*Pdhb*) mRNA in the SuHx group with the expression returning to control levels in the SuHx/ACTZ group. We did not find significant differences in the expression of other enzymes of the citric acid cycle including citrate synthase (*Cs*) and isocitrate dehydrogenase (*Idh2*) (Figure 7C). We found a similar pattern in the metabolic profile of the LV when we evaluated the expression of the above markers, but the changes were of a smaller magnitude (Supplementary Figure 6).

## Discussion

We report a distinct transcriptomic signature of RVH in the early phase of SuHx-induced PAH (3 weeks) that is suggestive of dysregulated metabolic pathways in association with RVH and fibrosis. We further demonstrate a favorable response to ACTZ treatment with prevention of RVH and fibrosis, improvement of RV compliance and function, and a shift toward normalization of the RV gene expression that suggests reversal of metabolic switching at the transcriptomic level.

The transcriptomic profile of the RV in the SuHx rat model of PAH was previously described by Legchenko et al. (29) who performed RNA-seq analysis at a much later time point (9 weeks) and used a 3-week-hypoxic group as control. At this late stage of RVF that was associated with increased mortality, the transcriptomic analysis along with complementary imaging modalities demonstrated inhibition of fatty acid transport and oxidation along with abnormal glucose uptake, mitochondrial dysfunction, and impaired oxidative phosphorylation (29). These findings are also in agreement with findings in humans with end-stage PAH (30). In our studies, we demonstrate a similar inhibition of fatty acid transport and oxidation at the transcriptomic level, at a much earlier time point, suggesting that these metabolic alterations occur early in the disease process and thus may be therapeutically targeted.

The contribution of RV metabolic reprogramming to PH-associated RVF was also demonstrated by Graham et al. (31) who described the vascular adaptation of the RV to experimental PH in the SuHx model in female rats at Denver altitude. Using stereology, these authors found significant augmentation of the RV vascular network in the PH animals. Additionally, using steady-state metabolomics at the 8-week time point, they demonstrated that the altered substrate utilization by the RV is not due to inadequate metabolic substrate delivery but rather due to an intrinsic RV metabolic switch in substrate utilization.

Drake et al. (32) evaluated the molecular signature of RVF using the SuHx model and pulmonary artery banding (PAB) + Cu^+2^-low diet model in rats and compared it to the adaptive RVH transcriptomic signature of the chronic hypoxic and PAB rat models. They used microarray and pathway analysis to conclude that the most significant changes in RVF were related to cell growth, angiogenesis, and energy metabolism. Importantly, they demonstrated that these changes were reversible with carvedilol treatment in the SuHx model. Another study by Gomez-Arroyo et al. (16) also tried to elucidate the metabolic dysregulation between adaptive and maladaptive RVH by comparing the RV metabolic profile of the PAB model and the SuHx model, respectively. The authors emphasized the impact of *Pgc-1*α downregulation on the transcriptional regulation of enzymes regulating beta oxidation during RVF. In this study, in which the length of hypoxic exposure after Sugen injection was 4, as opposed to 3 weeks in our study, a pattern of downregulated transcripts of acyl-CoA-dehydrogenases distinguished RVF from adaptive RVH in which acyl-CoA-dehydrogenase expression was increased. We observed the same pattern in our model at an earlier time point after 3 weeks of hypoxic exposure (decreased RV expression of *Pgc-1*α and acyl-CoA-dehydrogenases), and ACTZ treatment was associated with an increased expression similar to the control animals. This further supports our hypothesis that ACTZ has a favorable effect on RV dysfunction.

Furthermore, Potus et al. (33) reported on the transcriptomic signature of decompensated RVF in the monocrotaline (MCT) model and found changes in mitochondrial, inflammatory, and angiogenic abnormalities. Although our study lacks the detailed hemodynamic profiling of this prior study in the MCT model, the overall theme of mitochondrial–metabolic dysregulation is remarkably similar and corroborated by studies in human patients with end-stage PAH (30). A recent study by Murashige et al. (34) in patients with left heart failure provided definitive evidence that fatty acid utilization is the primary fuel source in normal human heart and that cardiac failure is accompanied by decreased fatty acid utilization. This study also supports an increased role for proteolysis in cardiac failure as well as increased utilization of ketones and lactate.

RV fibrosis was previously described in several models of PAH including the hypoxic (35), MCT (36), and SuHx models (37, 38), as well as in the PAB model that is not characterized by pulmonary vascular disease (38). Although initially adaptive, excessive RV fibrosis can impair RV function by various mechanisms including RV stiffness and diastolic dysfunction, impaired excitation–contraction coupling, disrupted coordination of myocardial contraction, and ventricular dilation (39). In agreement with prior studies (38, 40), we found significantly increased interstitial fibrosis in the RV in SuHx animals, whereas perivascular fibrosis was not increased. We found a significant decrease of RV fibrosis in the SuHx model of PAH in response to ACTZ treatment, and this was associated with improved echocardiographic markers of RV function. Other studies have reported similar findings with the use of the oral endothelin receptor antagonist bosentan in the hypoxic model (35), inhaled iloprost in the SuHx and PAB models (38), and the beta blocker bisoprolol in the MCT model (36). Additionally, da Silva Gonçalves Bós et al. (40) reported reversal of established PAH in the SuHx model with the use of the oral acetylcholinesterase inhibitor pyridostigmine. RV fibrosis is also a known feature of human PAH and is associated with RV dysfunction, but whether this is a causal and fully reversible process amenable to therapeutic targeting needs to be evaluated further (39).

Despite the beneficial effects we observed with ACTZ in our studies, the role of CAs in the pathogenesis of PH remains unclear. We have previously shown that CA II (CA2) is upregulated in the alveolar macrophages of SuHx animals at both the mRNA and protein levels that this coincided with macrophage activation. As a result, we found that ACTZ was associated with amelioration of pulmonary and systemic inflammation in the SuHx model (22). In addition, CA2 mRNA was increased in the lungs of SuHx animals compared to controls and in the lungs of human patients with idiopathic PAH. Given that CAs are ubiquitously expressed throughout the body, it is possible that ACTZ has pleiotropic effects as a CA inhibitor. In addition, ACTZ exerts physiologic effects that are independent of CA inhibition as reported by Shimoda et al. (41). Specifically in the heart, we speculate that ACTZ restores substrate utilization by promoting FAO, which has been shown to be beneficial as demonstrated by Legchenko et al. (29) with the use of *Ppar-*γ agonist pioglitazone. Additional studies are needed to further elucidate the molecular mechanisms underlying the protective effect of CA inhibition in RV function in PH. There are several limitations to our study. In our current study, ACTZ treatment was initiated 1 week into hypoxia, a time point when the PH phenotype of the SuHx model is not fully established (42); it should therefore be considered preventive. We previously showed that ACTZ ameliorated established disease when used at weeks 5–7 after SuHx, so it would be important to examine the RV metabolic profile at this later time point. Additionally, the dose and route of administration of ACTZ in this study (~100 mg/kg/day in drinking water) were based on prior work that demonstrated its effectiveness and tolerance in the experimental animals (22, 43). Other investigators have used doses of 10–200 mg/kg/day *via* the intraperitoneal route (44) indicating that the dose response and the most effective route of administration need to be further evaluated. Finally, our findings show a dysregulation at the gene expression level of the different metabolic pathways suggestive of a metabolic switch; however, further studies in cardiomyocytes are needed to elucidate the exact metabolic alterations in substrate utilization.

In conclusion, the unique RV transcriptomic signature of the SuHx model of PAH reveals a metabolic gene dysregulation with downregulation of transcripts that involve FAO. Our data support that this metabolic gene dysregulation occurs early in the disease process before the development of overt RVF. Treatment with ACTZ, a CA inhibitor, prevents PH and vascular remodeling with additional beneficial effects on the RV performance, crucial for patient outcomes. The improved RV function is associated with a partial restoration of the dysregulated metabolic profile including the expression of *Pgc1a* transcription factor, which is a key regulator of FAO and mitochondrial biogenesis. We suggest that the CA inhibitor ACTZ may exert pleiotropic beneficial effects in PAH that need to be further delineated in preclinical studies. In addition, given that ACTZ is currently in clinical trials for PAH, it is important to rigorously characterize its effects on cardiac function in order to leverage them therapeutically.

## Data Availability Statement

The datasets generated for this study can be found in online repositories. The names of the repository/repositories and accession number(s) can be found below: Gene Expression Omnibus under Accession GSE169652.

## Ethics Statement

The animal study was reviewed and approved by Brigham and Women's Institutional Animal Care and Use Committee.

## Author Contributions

FS designed and performed the study and participated in data collection, analysis, and manuscript writing. ZM participated in study execution, data collection, analysis, and manuscript editing. SV, KK, and PZ participated in study execution and manuscript editing. BF and BK analyzed RNA-seq data. SK provided input to study design, execution, and data analysis and reviewed the manuscript. HC contributed to study design, supervision of study execution, manuscript writing, data analysis, and final editing and approval of the manuscript. All authors contributed to the article and approved the submitted version.

## Conflict of Interest

The authors declare that the research was conducted in the absence of any commercial or financial relationships that could be construed as a potential conflict of interest.

## Supplementary Material

Supplementary Figure 1 Overview of the experimental design. Rats were injected with SU5416 (Sugen) or control DMSO and placed into hypoxia for 3 weeks. For the treatment group (SuHx+ACTZ); acetazolamide (ACTZ) was added to the drinking water on day 7.

Supplementary Figure 2 Principal component analysis (PCA) between SuHx and control groups.

Supplementary Figure 3 Heatmap across all samples of the significantly changed transcripts (FDR <0.05) involved in fatty acid oxidation. Featured scaling applied on normalized counts for presentation of the data. *n* = 5 individual animals in each group.

Supplementary Figure 4 Pulmonary Artery Acceleration Time (PAAT) quantitative data (*n* = 5–14 animals per group). Experimental groups: Control, SuHx, and SuHx+ACTZ. Statistical analysis by one-way ANOVA and Tukey's *post-hoc* test. Error bars are mean ± SEM (^*^*P* < 0.05, ^**^*P* < 0.01, and ns, non-significant).

Supplementary Figure 5 Treatment with ACTZ does not alter left ventricular performance and remodeling. **(A)** Ejection fraction. **(B)** Fractional shortening (FC). **(C)** Cardiac output (CO). **(D)** Left ventricular (LV) end diastolic volume. **(E)** Posterior wall thickness in diastole. **(F)** Anterior wall thickness in diastole. Quantitative data from 5 to 9 animals per group. Experimental groups: Control, SuHx, and SuHx+ACTZ. Statistical analysis by one-way ANOVA and Tukey's *post-hoc* test. Error bars are mean ± SEM.

Supplementary Figure 6 Effects of ACTZ treatment on LV metabolism associated markers in SuHx animals. **(A)** Fatty acid transport and oxidation transcripts. **(B)** Glucose uptake and tricarboxylic acid cycle (TCA) enzymes. **(C)** Mitochondrial transcription factors (*n* = 7–8 per group). Experimental groups: Control, SuHx, and SuHx+ACTZ. Statistical analysis by one-way ANOVA and Tukey's *post-hoc* test. Error bars are mean ± SEM (^*^*P* < 0.05 compared to control, ^#^*P* < 0.05 compared to SuHx).

Supplementary Table 1 Primers for rat genes in quantitative real-time PCR.
