# Supplementary figures and images for: Acetazolamide Improves Right Ventricular Function and Metabolic Gene Dysregulation in Experimental Pulmonary Arterial Hypertension
Source: Front Cardiovasc Med. 2021 Jun 17;8:662870. doi: 10.3389/fcvm.2021.662870 (PMC8247952; doi:10.3389/fcvm.2021.662870)

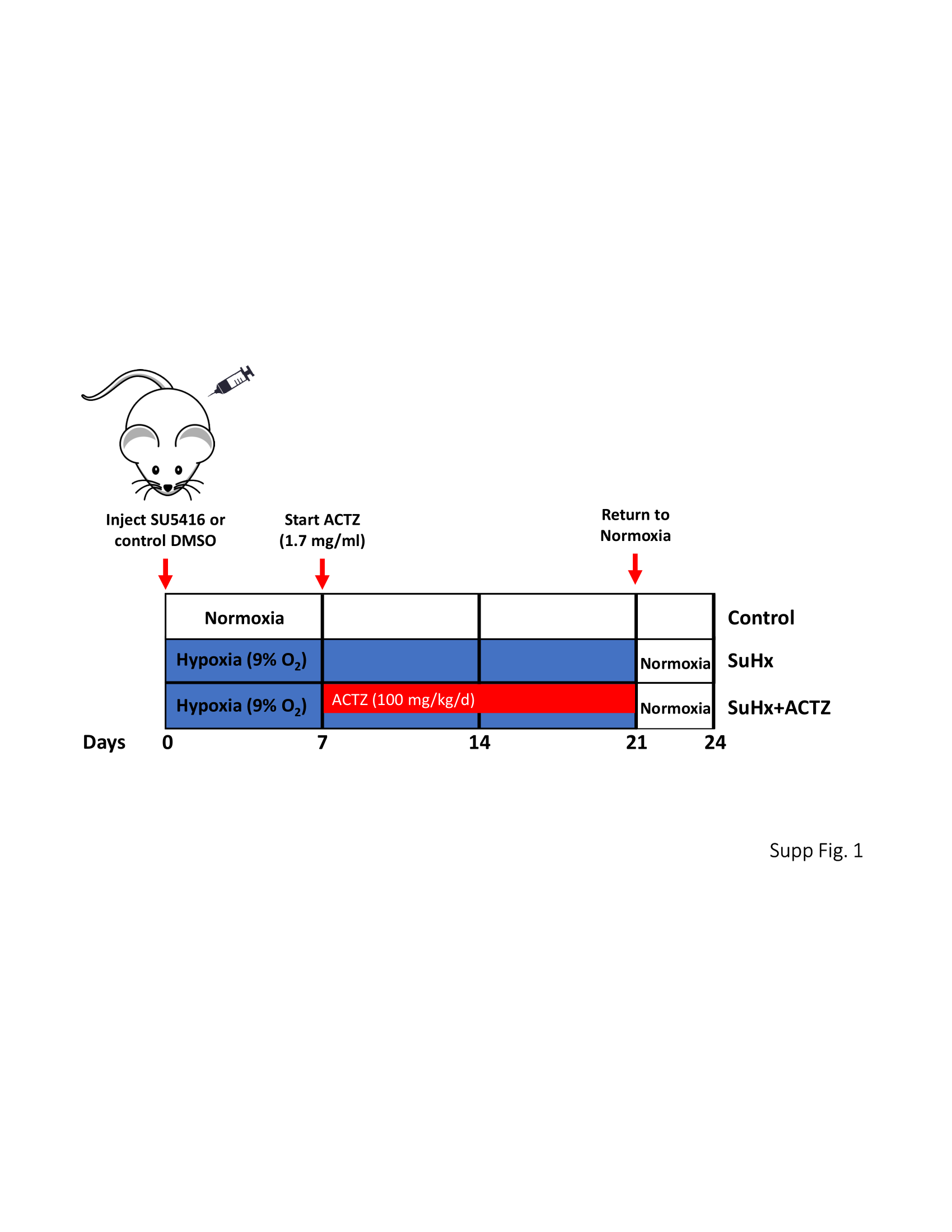

Supplement: Supplementary Figure 1 — Overview of the experimental design. Rats were injected with SU5416 (Sugen) or control DMSO and placed into hypoxia for 3 weeks. For the treatment group (SuHx+ACTZ); acetazolamide (ACTZ) was added to the drinking water on day 7. [file Image_1.TIFF]

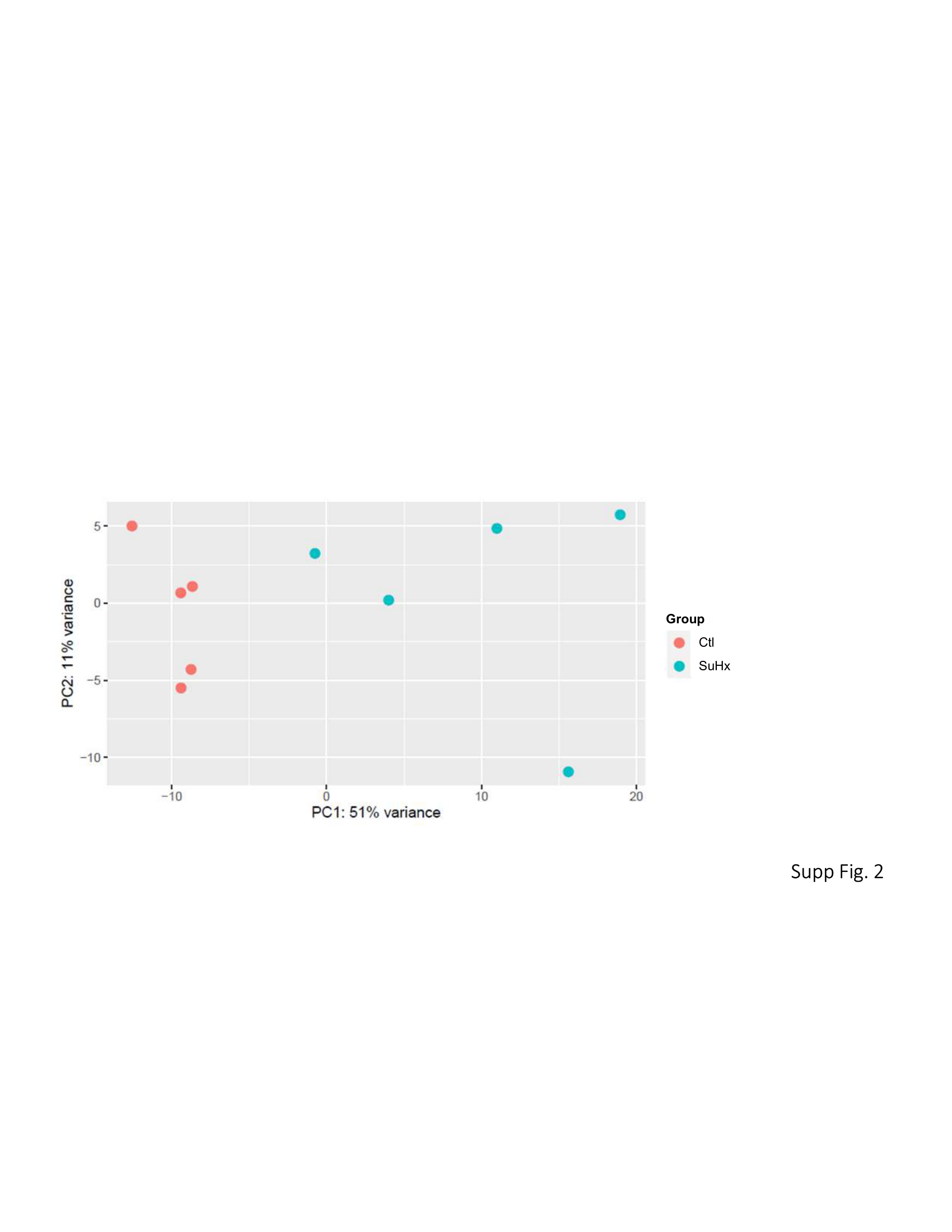

Supplement: Supplementary Figure 2 — Principal component analysis (PCA) between SuHx and control groups. [file Image_2.TIFF]

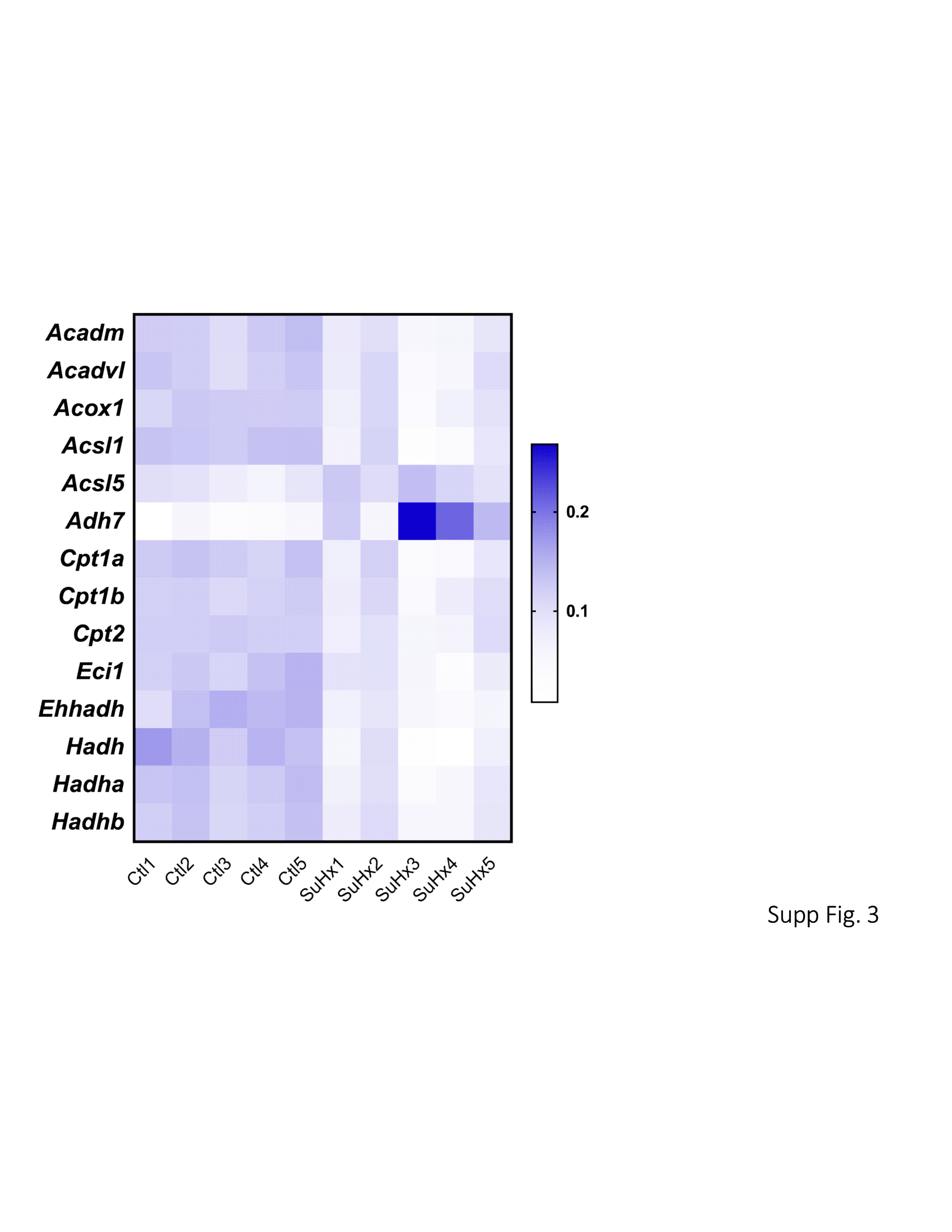

Supplement: Supplementary Figure 3 — Heatmap across all samples of the significantly changed transcripts (FDR <0.05) involved in fatty acid oxidation. Featured scaling applied on normalized counts for presentation of the data. n = 5 individual animals in each group. [file Image_3.TIFF]

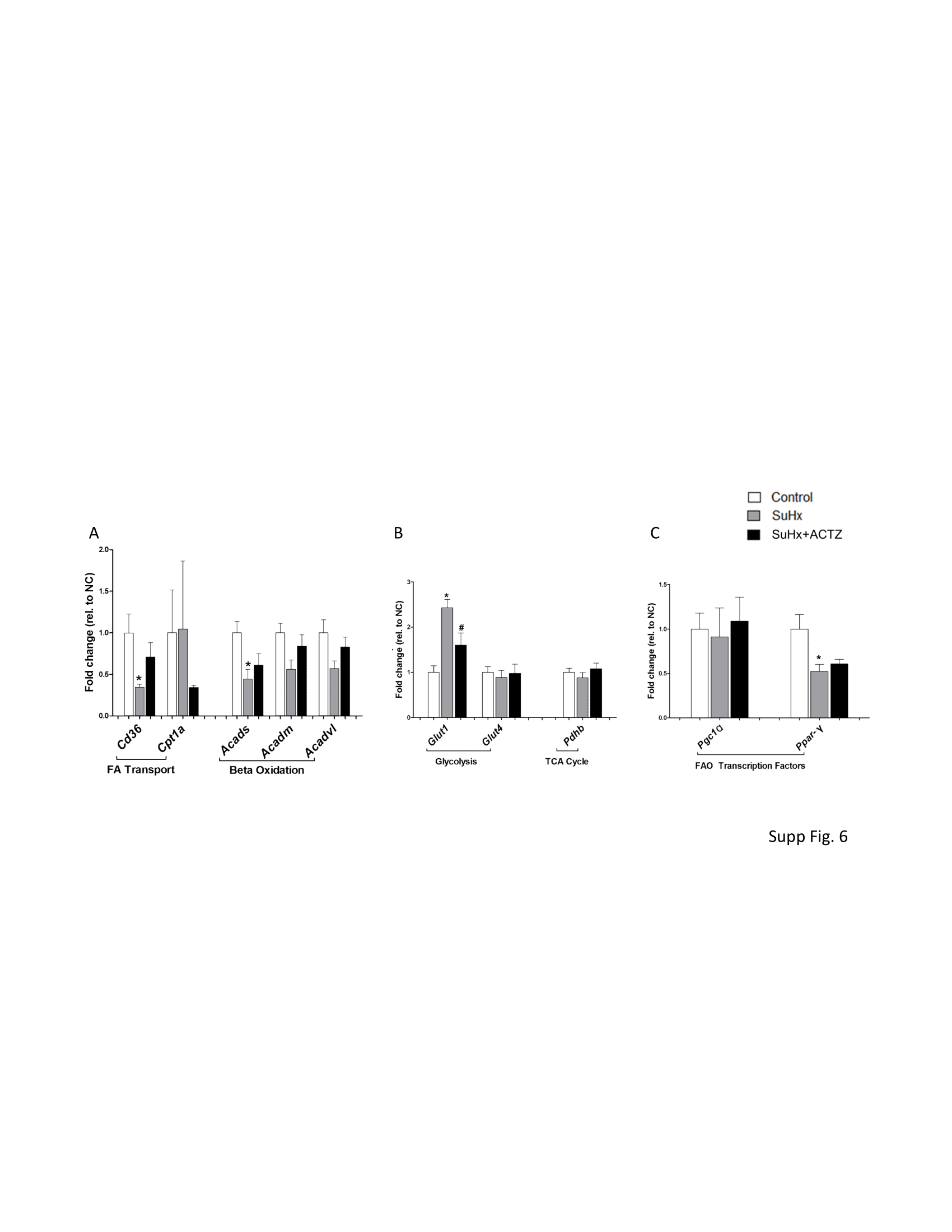

Supplement: Supplementary Figure 6 — Effects of ACTZ treatment on LV metabolism associated markers in SuHx animals. (A) Fatty acid transport and oxidation transcripts. (B) Glucose uptake and tricarboxylic acid cycle (TCA) enzymes. (C) Mitochondrial transcription factors (n = 7–8 per group). Experimental groups: Control, SuHx, and SuHx+ACTZ. Statistical analysis by one-way ANOVA and Tukey's post-hoc test. Error bars are mean ± SEM (*P < 0.05 compared to control, #P < 0.05 compared to SuHx). [file Image_6.TIFF]
