# Supplementary material for: Acetazolamide Improves Right Ventricular Function and Metabolic Gene Dysregulation in Experimental Pulmonary Arterial Hypertension
Source: Front Cardiovasc Med. 2021 Jun 17;8:662870. doi: 10.3389/fcvm.2021.662870 (PMC8247952; doi:10.3389/fcvm.2021.662870)
Supplement: Supplementary Table 1 — Primers for rat genes in quantitative real-time PCR. [file Table_1.docx]

**Supplementary Table 1.** Primers for rat genes in quantitative real-time PCR

| Gene | Accession No. | Forward Primer | | Reverse Primer |
| --- | --- | --- | --- | --- |
| *Ankrd2* | NM_001107589.1 | | 5'- CGGGATCCAGAACCTCATA -3' | 5'- CTTTCAGGAATGTCTCCTCATC -3' |
| *Acads* | NM_022512.2 | | 5’- GGGCCTCATCTACAGCTAA -3’ | 5’- CAGGGTTTGCATGGCTATC -3’ |
| *Acadm* | NM_016986 | | 5'- GGGTTTAGCTTCGAGTTGAC -3' | 5'- CAGGGTTTGCATGGCTATC -3' |
| *Acadvl* | NM_012891.2 | | 5'- CTAGGAGAAGTGGGAGATGG -3' | 5'- CATGATCAACCGCCTTGG -3' |
| *Cd36* | NM_031561.2 | | 5'- CATGCAAGTCCTGATGTCTC -3' | 5'- CAGTTATGGGTTCCACATCC -3' |
| *Col1a1* | NM_053304.1 | | 5'- GATGGATTCCAGTTCGAGTATG -3' | 5'- GCTGTTCTTGCAGTGATAGG -3' |
| *Col3a1* | NM_032085.1 | | 5'- GAACTCAAGAGCGGAGAATAC -3' | 5'- GTCATGGGACTGGCATTTAT -3' |
| *Cpeb1* | NM_001106276.1 | | 5'- GCTCTCTGAGTGTGGAGT -3' | 5'- CGGACGGACTTCTCTAGTT -3' |
| *Cpt1a* | [NM_031559.2](https://www.ncbi.nlm.nih.gov/nuccore/NM_031559.2) | | 5'- GCTGTATCGTCGCACATTAG -3' | 5'- TGGATGGTGTCTGTCTCTT -3' |
| *Cpt1b* | [NM_013200.1](https://www.ncbi.nlm.nih.gov/nuccore/NM_013200.1) | | 5’- GATGCAGTTCCAGAGAATCC -3’ | 5’- CACTCTACCCTTCCTCCTG -3’ |
| *Cs* | NM_130755.1 | | 5'- GCCAGAAACTGCTACCTAAG -3' | 5'- AAGAGACCTGTTCCTCTGT -3' |
| *Errα* | [NM_001008511.2](https://www.ncbi.nlm.nih.gov/nuccore/NM_001008511.2) | | 5'- TGGTGGTTGAACCTGAGAA -3' | 5'- GAGGAGAAGCCTGGGATG -3' |
| *Errγ* | NM_203336.2 | | 5'- AGGGTGGCAGATGAGTATAA -3' | 5'- GTGGAGAAGCCTGGGATA -3' |
| *Fam110c* | NM_001025051.1 | | 5'- AGGGTGGCAGATGAGTATAA -3' | 5'- TGGAGGTGGTAACATAGAGG -3' |
| *Fcgr3a* | NM_207603.1 | | 5'- CAGTCTATGAGGAACCCAAAC -3' | 5'- AGTCCACAGGGAAGCTAATA -3' |
| *Glut1* | NM_138827.1 | | 5'- TTCGGCTTAGACTCCATCA -3' | 5'- GAAGGGCAACAGGATACAC -3' |
| *Glut4* | NM_012751.1 | | 5'- CTCAATGGTTGGGAAGGAAA -3' | 5'- CCGTCCGAGAATGAGTATCT -3' |
| *Hk1* | [NM_012734.1](https://www.ncbi.nlm.nih.gov/nuccore/NM_012734.1) | | 5'- TGATGGAGGTGAAGAAGAGGC -3' | 5'- GGCAGCATTTTGACAGTAGCT -3' |
| *Hk2* | NM_012735.2 | | 5'- GCCGTAGTGGACAAGATAAG -3' | 5'- TGGCAAAGTGAGGATGAAG -3' |
| *Idh2* | [NM_001014161.1](http://www.ncbi.nlm.nih.gov/nuccore/NM_001014161.1) | | 5'- CACCAGTACCAACCCTATTG -3' | 5'- GAGTCTGTGCAAACCTGAT -3' |
| *Ldha* | [NM_017025.1](https://www.ncbi.nlm.nih.gov/nuccore/NM_017025.1) | | 5'- TCCCTGAAGTCTCTGAACC -3' | 5'- CACTTCGTATGCACTGTCAA -3' |
| *Ldhb* | [NM_012595.2](https://www.ncbi.nlm.nih.gov/nuccore/NM_001316333.1) | | 5'- TCCAACCCAGTGGATATTCT -3' | 5'- GGCCATGAGGTAACGAAAC -3' |
| *Nppa* | NM_012612.2 | | 5'- TTTCAAGAACCTGCTAGACC -3' | 5'- CTCAGAGAGGGAGCTAAGT -3' |
| *Nppb* | NM_031545.1 | | 5'- GATGATTCTGCTCCTGCTTT -3' | 5'- GAGCCATTTCCTCTGACTTT -3' |
| *Pdhb* | NM_001007620 | | 5’- GACAGTTCGTGAAGCCATTA -3’ | 5’- GCCTCTGCTAACCTTGTATG -3’ |
| *Ppargc1a* | NM_031347.1 | | 5'- GGGATGATGGAGACAGCTA -3' | 5'- TTCATTCGACCTGCGTAAAG -3' |
| *Pparα* | [NM_013196.1](https://www.ncbi.nlm.nih.gov/nuccore/NM_013196.1) | | 5'- GATTTCTCAGTCCCTCGGA -3' | 5'- AGAGAGGGTGTCTGTGATG -3' |
| *Pparγ* | [NM_013124.3](http://www.ncbi.nlm.nih.gov/entrez/viewer.fcgi?db=nucleotide&id=223941861) | | 5'- GTCTCACAATGCCATCAGG -3' | 5'- AGCAGACTCTGGGTTCAG -3' |
| *Tgfb1* | [NM_021578.2](http://www.ncbi.nlm.nih.gov/nuccore/NM_021578.2) | | 5'- AACAATTCCTGGCGTTACC -3' | 5'- CCCTGTATTCCGTCTCCTT -3' |

Definition of genes: *Ankrd2* (ankyrin repeat domain-containing protein 2), *Acads* (short-chain Acyl-CoA dehydrogenase), *Acadm* (medium-chain Acyl-CoA dehydrogenase), *Acadvl* (very long-chain Acyl-CoA dehydrogenase), *Cd36* (cluster of differentiation 36), *Col1a1* (collagen type I alpha 1 chain), *Col3a1* (collagen type III alpha 1 chain), *Cpeb1* (cytoplasmic polyadenylation element binding protein 1), *Cpt1a* (carnitine palmitoyltransferase 1A), *Cpt1b* (carnitine palmitoyltransferase 1B), *Cs* (citrate synthase), *Errα* (estrogen-related receptor alpha), *Errγ* (estrogen-related receptor gamma), *Fam110c* (family with sequence similarity 110 member C), *Fcgr3a* (Fc fragment of IgG receptor IIIa), *Glut1* (glucose transporter 1), *Glut4* (glucose transporter 4), *Hk1* (hexokinase 1), *Hk2* (hexokinase 2), *Idh2* (isocitrate dehydrogenase (NADP(+)) 2), *Ldha* (lactate dehydrogenase A), *Ldhb* (lactate dehydrogenase B), *Nppa* (natriuretic peptide precursor A), *Nppb* (natriuretic peptide precursor B), *Pdhb* (pyruvate dehydrogenase beta subunit), *Ppargc1a* (peroxisome proliferator-activated receptor gamma coactivator 1-alpha), *Pparα* (peroxisome proliferator-activated receptor alpha), *Pparγ* (peroxisome proliferator-activated receptor gamma), *Tgfb1* (transforming growth factor beta 1).
